# Energy Calibration of a CdTe Photon Counting Spectral Detector with Consideration of its Non-Convergent Behavior

**DOI:** 10.3390/s16040518

**Published:** 2016-04-11

**Authors:** Jeong Seok Lee, Dong-Goo Kang, Seung Oh Jin, Insoo Kim, Soo Yeol Lee

**Affiliations:** 1 Korea Electrotechnology Research Institute, Ansan, Gyeonggi 15588, Korea; js900331@gmail.com (J.S.L.); dgkang@keri.re.kr (D.-G.K.); sojin@keri.re.kr (S.O.J.); iskim@keri.re.kr (I.K.); 2 Department of Biomedical Engineering, Kyung Hee University, Yongin, Gyeonggi 17104, Korea

**Keywords:** photon counting spectral detector, spectral x-ray imaging, tube voltage, Medipix-2, CdTe

## Abstract

Fast and accurate energy calibration of photon counting spectral detectors (PCSDs) is essential for their biomedical applications to identify and characterize bio-components or contrast agents in tissues. Using the x-ray tube voltage as a reference for energy calibration is known to be an efficient method, but there has been no consideration in the energy calibration of non-convergent behavior of PCSDs. We observed that a single pixel mode (SPM) CdTe PCSD based on Medipix-2 shows some non-convergent behaviors in turning off the detector elements when a high enough threshold is applied to the comparator that produces a binary photon count pulse. More specifically, the detector elements are supposed to stop producing photon count pulses once the threshold reaches a point of the highest photon energy determined by the tube voltage. However, as the x-ray exposure time increases, the threshold giving 50% of off pixels also increases without converging to a point. We established a method to take account of the non-convergent behavior in the energy calibration. With the threshold-to-photon energy mapping function established by the proposed method, we could better identify iodine component in a phantom consisting of iodine and other components.

## 1. Introduction

Photon counting spectral x-ray detectors (PCSDs) are capable of identifying photon energy of an incoming x-ray photon, thereby making it possible to produce spectral images [1]. A detector element of a PCSD outputs an electrical pulse, upon arrival of an x-ray photon at the detector element, whose amplitude is proportional to the photon energy. Spectral x-ray imaging systems are considered to have many potential clinical applications such as differentiation of contrast agents [2,3,4], k-edge imaging [5], atherosclerotic plaque study [6], and quantitative soft tissue imaging [7]. Particularly in k-edge imaging in which k-edges are separated apart by only few keV in many biomaterials, accurate energy calibration is essential. To identify the photon energy accurately, energy calibration of the detector, *i.e.*, mapping of the output pulse amplitude onto the photon energy, is necessary. The energy calibration has been accomplished using monochromatic radiation sources of a known photon energy such as a synchrotron x-ray source [8], radio-isotopes [9,10,11], or x-ray fluorescent materials [12,13,14]. However, use of a monochromatic x-ray source for the energy calibration of every detector in mass production of x-ray imaging systems is not practical since most x-ray imaging systems are not easily configured to adopt monochromatic x-ray sources in them and it has a limitation of long measurement time [14,15].

Recently, a practical and efficient energy calibration method has been introduced in which the x-ray tube voltage is used as a reference for the energy calibration [15,16]. In this method, a global threshold of the comparators in the detector is found that makes 50% of the detector elements turned off at a given tube voltage, and then, the global threshold is supposed to be corresponding to the maximum photon energy determined by the peak tube voltage, that is, kVp. After finding thresholds that correspond to a few different tube voltages, a linear mapping function is found that best fits the relation between the thresholds and the maximum photon energies. With this method, an energy calibration can be made with an x-ray source and a PCSD placed at the actual imaging system without using any extra measuring devices. Therefore, the energy calibration can be made *in situ*, which is of great practical merits in developing or producing spectral x-ray imaging systems.

In single pixel mode (SPM) PCSDs in which every individual detector element outputs electric pulses upon arrival of x-ray photons, the amplitude of the electric pulse at a certain detector element is not solely determined by the energy of the x-ray photon that hits the detector element [17]. Other factors, such as charge sharing among neighboring detector elements upon arrival of a single x-ray photon [18] or pulse overriding upon arrival of multiple x-ray photons in a narrow time interval [19,20] or electronic noise called kTC noise [21], may complicate the relation between the pulse amplitude and the x-ray photon energy. Particularly in cadmium telluride (CdTe) PCSDs having a small pixel pitch and a thick CdTe layer, the charge sharing effects may degrade spectral resolution of the detector to a great extent [17].

In the energy calibration of a Medipix-2 CdTe PCSD using the x-ray tube voltage, we observed some dependency of the fraction of off pixels on the x-ray exposure time. For more accurate energy calibration, we developed an energy calibration method that takes account of this dependency. After calibrating the detector, we took k-edge images of an iodine phantom to validate the energy calibration method.

## 2. Methods

### 2.1. Choosing Good Pixels of a CdTe PCSD

In Bremsstrahlung radiation, the photon energy has a continuous distribution in which the maximum photon energy is determined by the peak tube voltage applied to the x-ray source. Since the amplitude of an electric pulse produced by an x-ray photon is largely proportional to the incoming x-ray photon energy, the x-ray detector will produce no output signals if the voltage threshold at the comparator of the detector is higher than the electric pulse produced by a photon with the maximum energy. Therefore, in a single-element detector, the energy calibration, that is, mapping the amplitudes of electric pulses onto the photon energies, can be made by finding a threshold that makes the output signal begin to disappear. Once the thresholds has been found for a few different tube voltages, a linear mapping function can be found with which thresholds are mapped onto photon energies. In a 2D detector, the energy calibration can be made faster if ergodicity of pixel responses can be assumed. If all the pixel responses are the same, the probability that a pixel produces no output signals will be 0.5 at the threshold that corresponds to the maximum photon energy. Therefore, by finding a threshold that makes half of pixels on a 2D detector turned off, the energy calibration can be made without using any extra radiation sources or instrumental devices.

We calibrated the energy of a CdTe PCSD (XRI-UNO, IMATEK, Barcelona, Spain) that has a matrix size of 256 × 256. The PCSD has a 1 mm thick CdTe layer and a pixel pitch of 55 μm [22,23,24]. The bias voltage over the CdTe layer was 350 V and the detector was cooled by a Peltier cooler. The room temperature was 23~24 °C. The detector is based on Medipix-2 readout chip and each pixel has a comparator to produce a pulse if the preamplifier output is higher than the threshold voltage (threshold DAC hereafter) produced by a 10 bit digital-to-analog converter (DAC). Each pixel has an additional 4-bit digital-to-analog converter (DAC) to compensate the inhomogeneity of detector pixel responses and a 14-bit counter for photon counting. For x-ray generation, we used a micro-focus x-ray source (L8121-01, Hamamatsu, Iwata, Japan) that has a focal spot size of 5~50 μm depending on the operating tube power. The maximum tube voltage and tube current are 150 kVp and 500 μA, respectively. We operated the x-ray source in a continuous mode with a source-to-detector distance (SDD) of 575 mm with additional filtration by 2 mm thick aluminum plate.

**Figure 1:**
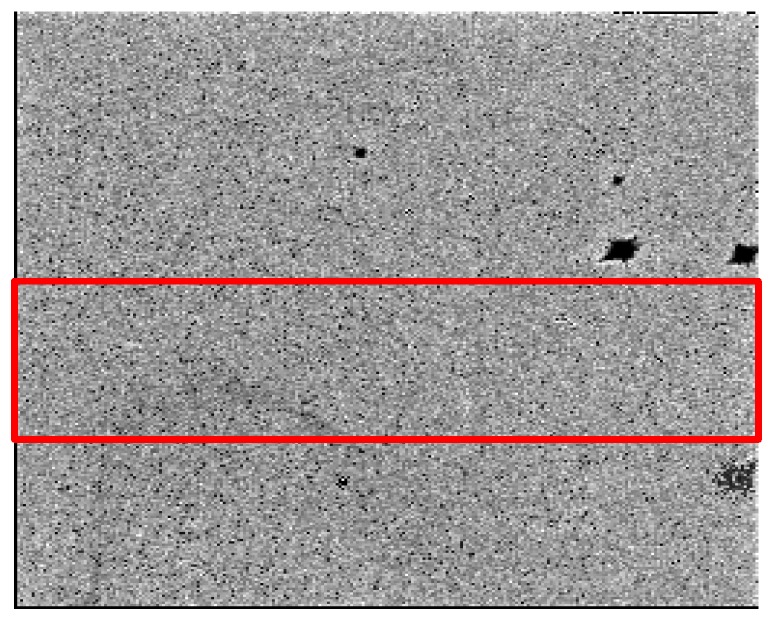
An x-ray image of air acquired by the CdTe photon counting spectral detector at 60 kVp, 217 μA. The exposure time was 1 s. Only good pixels in the red-marked region were used for the energy calibration avoiding the dead pixel clusters.

**Figure 2:**
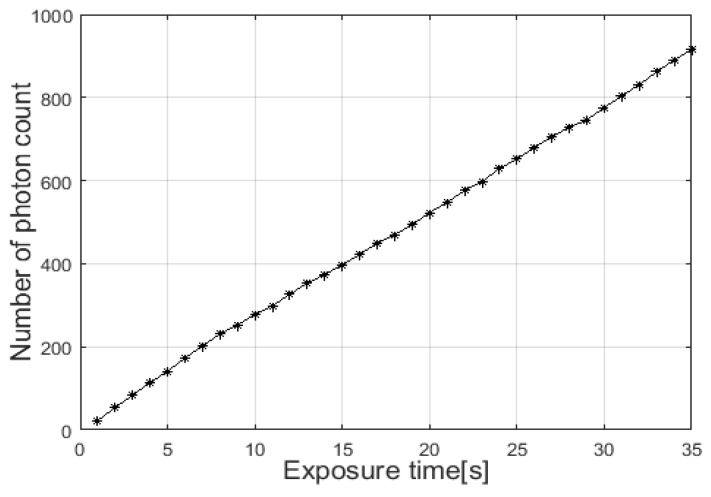
An example of the pixel responses to the exposure time. Only the pixels that have *R*^2^ value bigger than 0.999 at the linear fitting were used for the energy calibration.

Figure 1 shows an image acquired by the detector when nothing is placed between the x-ray source and the detector. The x-ray tube voltage was 60 kVp, the tube current was 217 μA, the exposure time was 1 s, and the detector temperature was 24.7 °C. As can be noticed from the figure, there are many dead pixels and dead pixel clusters. The appearance of the dead pixels is attributed to imperfections of the bump bonding and the internal crystal structure. To exclude the dead pixel clusters in the energy calibration, we chose good pixels inside the red-marked region in Figure 1. The red-marked region amounts to 27% of all the pixels. To choose good pixels, we obtained x-ray images of nothing with increasing the exposure time from 1 s to 35 s with the tube voltage and current of 60 kVp and 217 μA, respectively. Then, we applied linear fitting of the photon counts with respect to the exposure time at each pixel. Figure 2 shows an example of detector response at a good pixel. If *R*^2^ in the linear fitting is greater than 0.999 at a pixel, the pixel was classified as a good pixel. In the red-marked region, about 96% of pixels were classified as good pixels. In the energy calibration, only the good pixels were taken into account.

To see the noise characteristics of the good pixels, we took 400 images of air with an exposure time of 1 s and threshold DAC of 600. At the image acquisition, we set the tube voltages and tube currents in a way that equalizes area of the spectra. After taking the images, we computed mean and variance of the photon counts at the good pixels. As a result, all good pixels show the Poisson noise characteristics of the photon counts of which the mean and variance at a given tube voltage are equal to each other. Table 1 summarizes the mean and variance of the photon counts at a good pixel (168, 179) in several spectra.

### 2.2. Observation of Exposure Dependency of OFF Pixel Fraction

**Figure 3:**
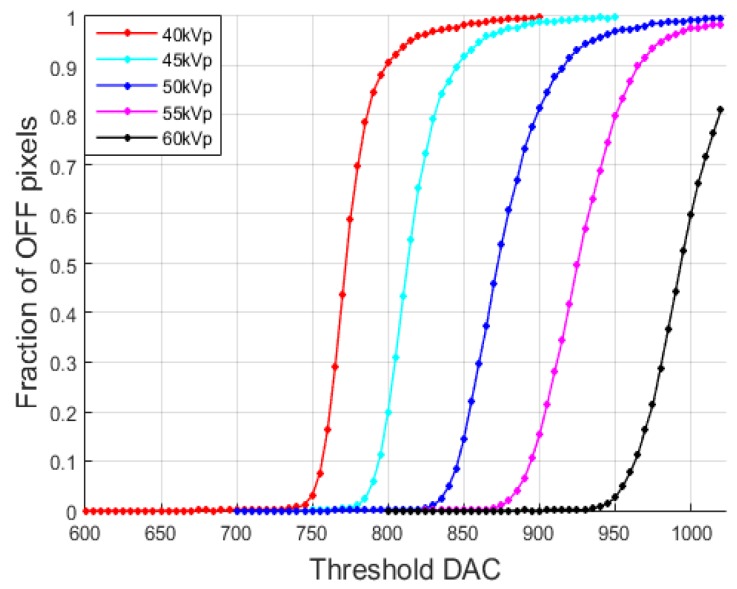
Fraction of OFF pixels with respect to the threshold DAC observed with the tube voltages of 40, 45, 50, 55 and 60 kVp. The tube current was 200 μA and the exposure time was 20 s.

After increasing the threshold DAC step by step at a given tube voltage and tube current, we computed the fraction of OFF pixels among the good pixels. Figure 3 shows the fraction with respect to the threshold DAC when the tube voltages were set to 40, 45, 50, 55 and 60 kVp at the tube current of 200 μA and the exposure time of 20 s. The detector temperature was 24~27 °C.

**Figure 4:**
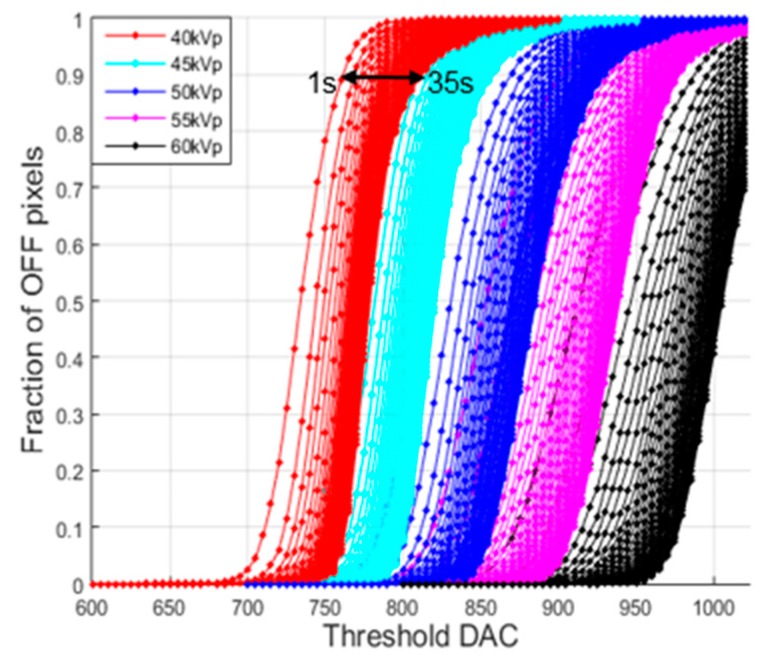
The fraction of OFF pixels with respect to the threshold DAC observed with the tube voltages of 40, 45, 50, 55 and 60 kVp. The tube current was 200 μA and the exposure time was varied from 1 s to 35 s.

At low exposure time, the fraction of OFF pixels, at around the threshold DAC where 50% pixels turned from off to on, would decrease as the exposure time increased since the probability of arrival of at least a single x-ray photon having the maximum energy at one of the pixels will increase. Therefore, the transition threshold, the threshold DAC corresponding to 50% fraction, will move to the right. That is, higher thresholds are necessary at higher exposures. However, the transition threshold was expected to converge to some point as the exposure time increases. Figure 4 shows the fraction of OFF pixels with respect to threshold DAC when the exposure time is increased from 1 s to 35 s at the tube current of 200 μA and the tube voltages of 40, 45, 50, 55 and 60 kVp. The detector temperature was 24~27 °C. As can be seen from the figure, the transition curves shift to the right as exposure time increases, and the shift is faster at higher tube voltages. Even though the shift to the right is slowing down as the exposure time increases, the shift never reaches a convergence point within the exposure time of 35 s.

**Figure 5:**
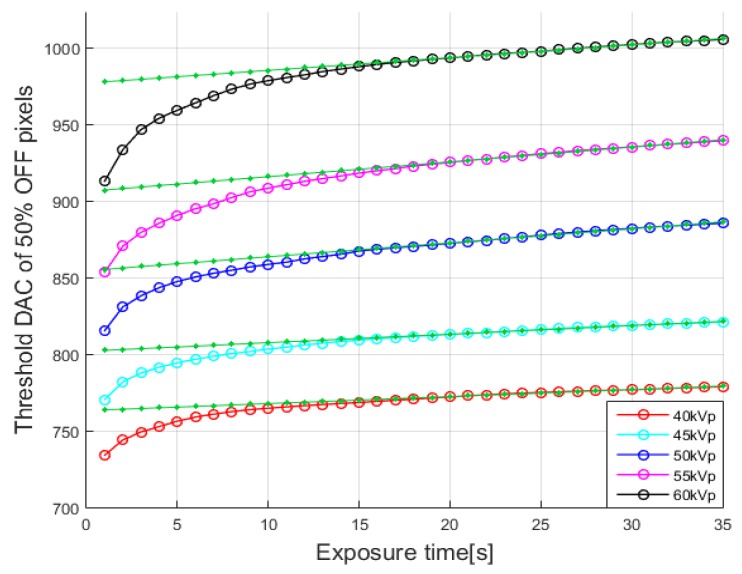
The transition threshold corresponding to 50% OFF pixel at 40, 45, 50, 55 and 60 kVp and at 200 μA. The green lines are linear fits to the parts of the curves after 15 s.

Figure 5 shows the thresholds DAC corresponding to 50% OFF pixel fraction with respect to the exposure time. In all the cases of tube voltages, 40, 45, 50, 55 and 60 kVp, we can observe a slow but steady increase of threshold DAC with respect to the exposure time. This is against the expectation that the detector would never produce a counting pulse when the threshold DAC is higher than the one corresponding to the maximum photon energy. The possible causes of this phenomenon would be sensitive detector response to the external environment variation, kTC noise of the detector, pileup effect, and so on. Among these, we attribute the steady increase of threshold DAC to kTC noise and pileup effect as the main reason. kTC noise is thermal noise on capacitors, and pileup effect is coincident pulse formation by multiple photons arriving almost simultaneously [19,20,21]. Since we took account of thresholds giving 50% pixel OFF, any single counting pulse, whose height is bigger than the original height due to some perturbations such as pileup effect and noise, will increase the number of ON pixels.

**Figure 6:**
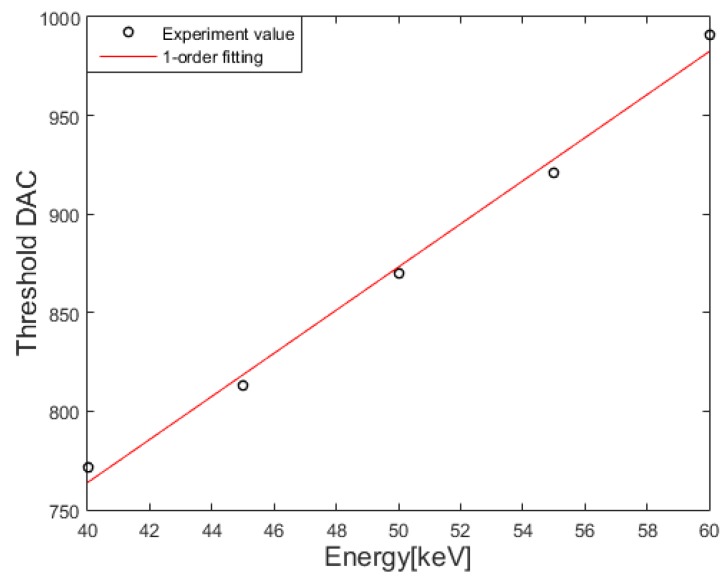
The derived mapping function between the photon energy and the threshold DAC.

The number of photon counts produced by noise and pileup effects will be proportional to the exposure time. Therefore, we thought that the threshold DAC corresponding to 50% fraction of OFF pixels would increase linearly with respect to the exposure time once the threshold DAC exceeds the one corresponding to the maximum photon energy, so we applied linear fitting to the parts of the curves after 15 s as shown by the green lines in Figure 5. We found that the first crossing points between the linear fitting lines and the threshold DAC curves were 22 s at 40, 60 kVp, 23 s at 45, 55 kVp, and 24 s at 50 kVp. So, we set the exposure time to the first crossing points between the linear fit and the threshold DAC curve to derive a mapping function at 200 μA. We did not choose an exposure time after the crossing points since the slow increase of threshold DAC was attributed to the noise and pileup effect. We did not choose an exposure time too much earlier than the crossing points either since the number of photon counts was not statistically sufficient to conclude 50% pixel OFF. The linear mapping function derived from the threshold DAC of 50% OFF pixel, at the exposure times of 22 s at 40, 60 kVp, 23 s at 45, 55 kVp, and 24 s at 50 kVp, is:

(1) TH_DAC=11.176×E+320.13

where E is the photon energy in keV and TH_DAC is the threshold DAC. In the linear fitting, *R*^2^ values was 0.995 and root mean square deviation was 5.79 in threshold DAC. Figure 6 shows the linear mapping function from which the iodine k-edge of 33.17 keV corresponds to 691 in threshold DAC.

### 2.3. k-Edge Imaging of Iodine

**Figure 7:**
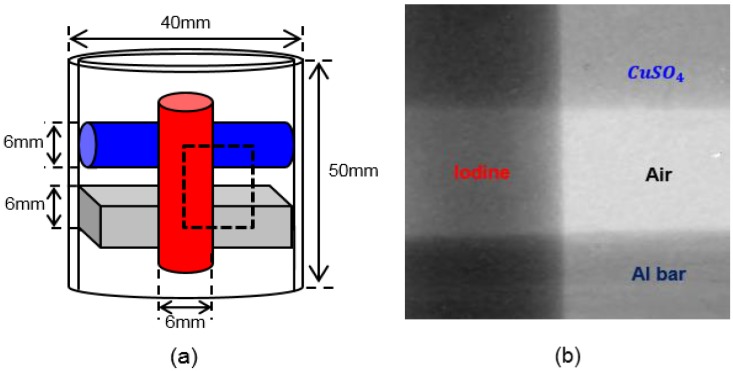
(**a**) A schematic illustration of the phantom consisting of two tubes filled with iodine solution and CuSO_4_ solution, respectively, and an aluminum bar. (**b**) A projection image of the phantom taken with a field of view marked as a dashed box at 60 kVp, 200 μA, 15 s.

To validate the mapping function, we performed k-edge imaging of an iodine phantom. We made a phantom consisting of 0.5 M iodine solution, 1.5 M CuSO_4_ solution and an aluminum (Al) bar. The iodine and CuSO_4_ solution was filled in acrylic tubes of 6 mm diameter and 30 mm height, and the Al bar had a size of 6 mm × 6 mm × 30 mm. The CuSO_4_ tube and the Al bar are glued to the iodine tube as shown in Figure 7. The tubes and bar were supported by an acrylic cylinder of 40 mm diameter, 50 mm height and 3 mm thickness. Figure 7 shows details of the phantom with a projection image of it. The projection image was taken with the field of view (FOV) marked by the dashed box in the schematic of the phantom. On the projection image of the phantom shown in the right-hand side, iodine, CuSO_4_ and aluminum are overlapped in some regions as indicated in the figure. In some regions, there is a single component, either iodine or CuSO_4_ or aluminum. In the overlapped region, we tried to separate the iodine component from other components.

After the energy calibration, we took projection images of the phantom with changing threshold DAC from 600 to 800 with a step size of one at the tube voltage of 60 kVp and the tube current of 200 μA. In the case of a single contrast agent imaging, the normalized expectation value of number of photon counts in a certain energy bin will be represented by [5,24,25]:

(2) $$N_{i}=-\text{log}[\frac{\int_{0}^{\infty }S_{i}(E)\Phi (E)e^{-\sum_{j=1}^{3}f_{j}(E)A_{j}}D(E)dE}{\int_{0}^{\infty }S_{i}(E)\Phi (E)D(E)dE}]$$

in which *A_j_* is the line-integral of the basis-material densities along a geometrical line connecting the x-ray source and the detector, S_*i*_(E) is the bin-sensitivity function (of the *i*-th energy bin whose bandwidth is 5 keV in this study). The index *i* is 1,…,N with N being the total number of energy bins. Φ(E) is the incident X-ray spectrum, f_*j*_(E) is the *j*-th attenuation basis functions, and *D*(E) is the detector absorption efficiency.

We define energy contrast as below [26]:

(3) $$C_{i}=N_{i}-N_{i+1}$$

in which C_*i*_ represents the difference of projection data between two nearby energy bins, hence, it somehow demonstrates contrast change along the energy direction. We compute C_*i*_ on some points in the phantom image, at which only one component, either iodine, CuSO_4_, or aluminum exists, to evaluate accuracy of the mapping function. 

## 3. Results

**Figure 8:**
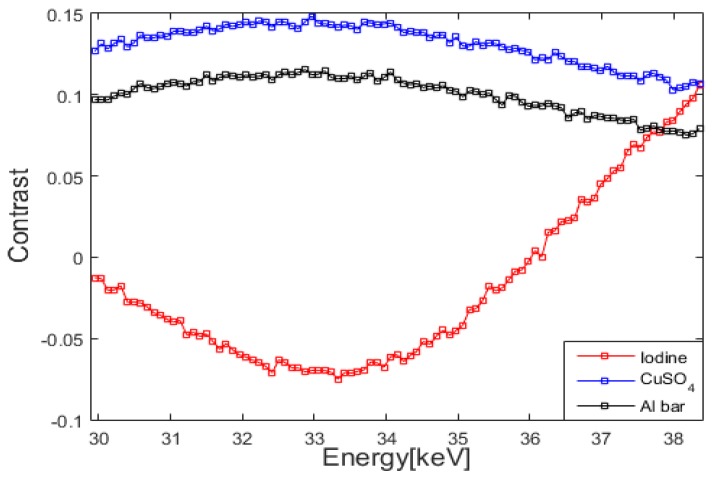
The derivatives of the projection images at the regions of iodine, CuSO_4_, and aluminum.

Figure 8 shows the energy contrast C_*i*_ at the points of iodine, CuSO_4_, and aluminum on the phantom image. At the chosen points, only one component exists without any overlapping of multiple components. In the case of iodine, the maximum contrast between iodine and other components appears at 33.27 keV which is very close the k-edge of iodine, 33.17 keV. The energy contrast of CuSO_4_ and aluminum changes much slower than that of iodine since there is no k-edges for them at the energy band.

**Figure 9:**
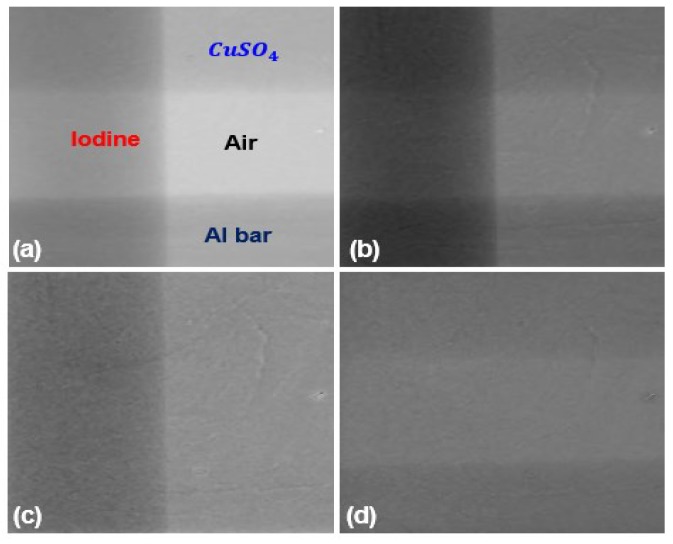
Spectral images. (**a**) A low band image between 24.9–33.27 keV. (**b**) A high band image between 33.27–42.2 keV. (**c**) An iodine image. (**d**) An image of CuSO_4_ and aluminum.

Figure 9 shows spectral images of the phantom. The image was taken with a tube voltage and current of 60 kVp and 200 μA with 2 mm thick aluminum filter. The exposure time was 15 s. We took three images of the phantom with threshold DACs corresponding to 24.9 keV, 33.27 keV and 42.2 keV. To alleviate dead pixel effects, we applied 3 × 3 median filtering to every image of different threshold DAC. Figure 9a is a low band image covering the photon energy from 24.9 keV to 33.27 keV, and Figure 9b is a high band image covering from 33.27 keV to 42.2 keV. Figure 9c shows an iodine image made by weighted subtraction of the low band image (I_low_) and the high band image (I_high_) [27], that is, I_low_ − 1.5 × I_high_. Figure 9d shows an image of CuSO_4_ and aluminum made by I_low_ − 0.9 × I_high_.

## 4. Discussion

**Figure 10:**
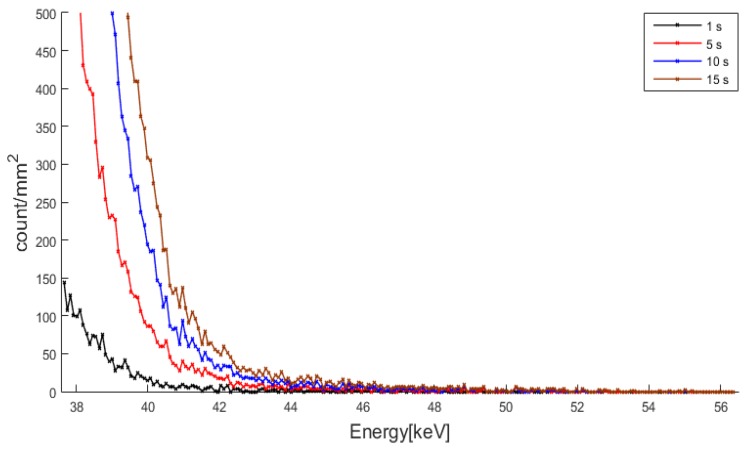
The energy spectra at 40 kVp and 200 μA. The photon counts on 1 mm^2^ are appearing above 40 keV because of the noise and pileup effect.

We attributed the non-convergent behavior of the detector to the kTC noise and pileup effect, and we obtained the mapping function with the exposure time before the noise and pileup effect begin to dominate. As the exposure time increases, the probability of receiving a counting pulse with higher amplitude than its original will increase, which in turn will increase the number of turned on pixels at the energy threshold higher than the maximum photon energy. To validate this explanation of the noise and pileup effect, we measured energy spectra at 40 kVp and 200 μA as the exposure time increases. Figure 10 shows the energy spectra, represented by number of photon counts on 1 mm^2^. As can be seen from the figure, the photon count keep appearing above the maximum photon energy, 40 keV, until around 55 keV. In between 40 keV and 55 keV, the photon count increases as the exposure time increases, suggesting that the noise and pileup effect produce counting pulses with higher amplitude than the one determined by the tube voltage. Therefore, the noise and pileup effect must be discarded in determining the mapping function.

We made mapping functions with different exposure times as well. Table 2 shows the iodine k-edge energies determined by the mapping functions which were derived with the exposure time of 1 s and 30 s. If the exposure time is too short, fewer pixels, than would be at the right threshold DAC, will be turned on due to insufficient influx of x-ray photons. If the exposure time is too high, more pixels will be turned on due to the noise and pileup effect. As compared to the actual iodine k-edge energy, too short or too long exposure times results in bigger errors, 7.36% and 1.78%, respectively.

Our experiments have some limitation in that we did not perform the energy calibration with a mono-energetic source. With a mono-energetic source, we could characterize the energy resolution of the detector, hence, we may better analyze the non-convergent behavior. However, it is known that single pixel mode PCSDs with fine pixel pitches do not show clear energy resolution [15]. Despite the limitation, the spectral images of the iodine phantom demonstrated the efficacy of the proposed calibration method. In conclusion, we have shown that accuracy of the energy calibration of photon counting detectors can be improved by taking the non-convergent behavior into account when the energy calibration is made by finding the threshold DAC which results in 50% pixel OFF.

## Figures and Tables

**Table 1:** Mean and variance of the photon counts at a good pixel in 400 measurements. The exposure time was 1 s and the threshold DAC was 600.

Tube Voltage (kVp)	Tube Current (μA)	Mean of Photon Counts	Variance of Photon Counts
40	490	232.0	252.2
50	313	395.6	419.7
60	217	570.0	582.4
70	160	735.5	796.1
80	122	892.0	843.8

**Table 2:** Iodine k-edge energies estimated from the mapping functions derived with different exposure times.

Exposure Time in Deriving Mapping Function	Estimated Iodine k-Edge (keV)	Error (%)
1 s	35.61	7.36
30 s	32.58	1.78
22 s at 40, 60 kVp	33.27	0.48
23 s at 45, 55 kVp		
24 s at 50 kVp		

## Abbreviations

The following abbreviations are used in this manuscript:

PCSD: Photon counting spectral detector
SPM: Single pixel mode
CSM: Charge summing mode
DAC: Digital to analog converter
FOV: Field of view
